# Obesity-Related Metabolic Dysfunction in Dairy Cows and Horses: Comparison to Human Metabolic Syndrome

**DOI:** 10.3390/life11121406

**Published:** 2021-12-16

**Authors:** Zsofia Daradics, Cristian M. Crecan, Mirela A. Rus, Iancu A. Morar, Mircea V. Mircean, Adriana Florinela Cătoi, Andra Diana Cecan, Cornel Cătoi

**Affiliations:** 1 Department of Pathology, Faculty of Veterinary Medicine, University of Agricultural Sciences and Veterinary Medicine, Mănăștur St. 3-5, 400372 Cluj-Napoca, Romania; sofia.daradics@usamvcluj.ro (Z.D.); cornel.catoi@usamvcluj.ro (C.C.); 2 Department of Anaesthesiology and Surgery, Faculty of Veterinary Medicine, University of Agricultural Sciences and Veterinary Medicine, Mănăștur St. 3-5, 400372 Cluj-Napoca, Romania; cristian.crecan@usamvcluj.ro; 3 Department of Reproduction, Obstetrics and Veterinary Gynaecology, Faculty of Veterinary Medicine, University of Agricultural Sciences and Veterinary Medicine, Mănăștur St. 3-5, 400372 Cluj-Napoca, Romania; mirela.tripon@usamvcluj.ro (M.A.R.); iancu.morar@usamvcluj.ro (I.A.M.); 4 Department of Internal Medicine, Faculty of Veterinary Medicine, University of Agricultural Sciences and Veterinary Medicine, Mănăștur St. 3-5, 400372 Cluj-Napoca, Romania; 5 Department of Pathophysiology, Faculty of Medicine, “Iuliu Hatieganu” University of Medicine and Pharmacy, Victor Babeș St. 3-4, 400012 Cluj-Napoca, Romania; andra.cecan@umfcluj.ro

**Keywords:** obesity, laminitis, insulin resistance, insulin dysregulation

## Abstract

Obesity has become a serious health problem with frequent occurrence both in human and animal populations. It is estimated that it may affect over 85% of the human population and 70–80% of horses and cows by 2030. Fat cow syndrome (FCS) is a combination of metabolic, digestive, infectious, and reproductive disorders that affects obese periparturient dairy cows, and occurs most frequently in loose-housing systems, where periparturient and dry cows are fed and managed in one group disregarding the lactation stages. Equine metabolic syndrome (EMS) was named after human metabolic syndrome (MetS) and has insulin dysregulation as a central and consistent feature. It is often associated with obesity, although EMS may occur in a lean phenotype as well. Other inconsistent features of EMS are cardiovascular changes and adipose dysregulation. Laminitis is the main clinical consequence of EMS. MetS holds a 30-years old lead in research and represents a clustering of risk factors that comprise abdominal obesity, dyslipidemia, hypertension, and hyperglycemia (impaired fasting glucose or type 2 diabetes mellitus—T2DM), which are associated with doubled atherosclerotic cardiovascular disease risk, and a 5-fold increased risk for T2DM. The main aim of this review is to provide critical information for better understanding of the underlying mechanisms of obesity-related metabolic dysfunction in animals, especially in cows and horses, in comparison with MetS. Human medicine studies can offer suitable candidate mechanisms to fill the existing gap in the literature, which might be indispensable for owners to tackle FCS, EMS, and their consequences.

## 1. Introduction

Obesity is a major concern worldwide due to its increasing prevalence that can be ascribed to the availability of cheap foods, technological advancement, and built environments [1,2,3]. Obesity and insulin resistance (IR) are considered as the main underlying risk factors for metabolic disturbances and are involved in the rise of other risk factors, such as hypertension, hyperglycemia, and dyslipidemia [3,4,5,6]. The cluster of such risk factors is referred to as metabolic syndrome, a common condition among both human and animal populations [6,7]. Obesity is one of the most frequent characteristics of people with metabolic syndrome, and a high prevalence of visceral and abdominal obesity has been reported in this population [8,9,10,11]. In the setting of caloric overload, abdominal obesity and insulin resistant adipose tissue induce lipolysis, the release of great amounts of free fatty acids that cause hepatic IR in the liver. Subsequently, this disturbance induces increased gluconeogenesis, and, ultimately, hyperglycemia, cardiovascular disease, and type 2 diabetes mellitus (T2DM) [12,13,14].

Dairy cows are one of the most important sources of milk, and cow milk is used for the preparation of various dairy products indispensable for the human diet. Increasing demands induced long-term genetic selection of cows towards higher milk production [15]. However, this high genetic performance could only be reached when the cows are managed under optimal growth conditions. Overconditioning during the dry period, and overconsumption of energy at calving represent significant risk factors for modern dairy cows to develop numerous health problems during the transition period [15,16]. The transition period from 3 weeks before to 3 weeks after parturition is critically important for health, production, and profitability of dairy cows [17].

In horses, obesity can be defined as a pathological accumulation of fat which can cause adverse health problems. Conversely, many farmers deem a degree of obesity being normal, acceptable, or even desirable. For some agribusiness activities, horses are evaluated by their physical traits, so a degree of obesity is often judged to be an advantage on the field. If we look back to equine history, these species have been evolved as freely wandering independent species traversing wide areas on a daily basis to find a sufficient and suitable quantity of forage. Today, most domesticated horses suffer from physical inactivity, and their feed rations are drastically excessive with regards to their nutritional energy requirements. In addition, the forage sources for horses, such as pastures, paddocks, or hay used for feeding, contain genetically improved grassland species with enhanced nutritious content (https://www.safergrass.org, accessed on 7 September 2021), rich in sugars and starch. These risk factors are linked with the higher laminitis rates, and the development of insulin dysregulation (ID) (abnormal insulin metabolism) in horses [18,19,20]. Moreover, epidemiological studies identified several metabolic factors (low plasma adiponectin level, high serum insulin level, elevated body condition scores, presence of generalized or regional adiposity) as risk factors for the development of laminitis [21,22,23].

Although there are numerous differences between metabolic dysregulation in animals and humans in terms of clinical manifestations, complications, outcomes, etc., a number of disease mechanisms common in both species can be identified (e.g., root causes of metabolic syndrome, role of liver malfunction). This paper is a narrative review that aimed to discuss the underlying mechanisms of obesity-related metabolic dysfunction in cows and horses as compared to human metabolic syndrome (MetS).

## 2. Human Metabolic Syndrome (MetS)

The existence of a cluster of metabolic disturbances was described by Kylin in 1923, reporting the association of hyperuricemia, hyperglycemia, and hypertension [24]. Later, in 1947, Vague described two types of obesity: lower body adiposity, and abdominal adiposity, the latter being associated with cardiovascular disease and T2DM [25]. In 1988, Reaven linked this above-mentioned cluster of metabolic disturbances to insulin resistance, and termed it as Syndrome X [26]. One year later, Kaplan named the combination of upper-body obesity, glucose intolerance, hypertriglyceridemia, and hypertension as ‘The Deadly Quartet’ [27].

The adipose tissue has the central role in the pathophysiology of the syndrome by producing several bioactive molecules named adipokines [9,28]. Obese people are exposed to an increased concentration of circulating non-esterified fatty acids (NEFAs). These conditions induce a pro-inflammatory state, cardiovascular damage, and IR of the adipose tissue, liver, and skeletal muscle. Hypertrophic adipocytes become hypoxic and eventually necrotic, and lead to macrophages infiltration and activation, and to the overproduction of pro-inflammatory cytokines (tumor necrosis factor-alpha [TNF-α], interleukin-6 [IL-6], interleukin 1 [IL-1]) [29,30]. The continuous delivery of NEFAs to the liver induces ectopic fat, and brings about the installation of nonalcoholic fatty liver disease (NAFLD) [9,12]. Moreover, NEFAs accumulated in the liver lead to atherogenic dyslipidemia, i.e., hypertriglyceridemia, increased levels of low density lipoprotein (LDL), and decreased concentrations of high density lipoprotein (HDL) [13]. Beside hepatic functions, dyslipidemia has also been associated with postprandial phenomenon [31,32]. The development of hypertension is induced via several pathophysiological mechanisms, such as loss of vasodilator effect of insulin, increased secretion of angiotensinogen in the adipose tissue, and activation of the sympathetic nervous system and the renin–angiotensin–aldosterone system, associated with the increased renal absorption of sodium, and the expansion of the blood volume [6].

Several diagnostic criteria have been proposed by different organizations to define the combination of risk factors that defines HMS. The first working definition for MetS was released in 1998 by the World Health Organization (WHO), and was focused on glucose metabolism markers, considering IR the major underlying risk factor, plus at least any two of the following additional factors: obesity, impaired glucose regulation, hypertension, hypertriglyceridemia and/or low HDL cholesterol, and microalbuminuria [33]. One year later, in 1999, the European Group for Study of Insulin Resistance (EGIR) proposed the term of IR syndrome instead of MetS, as the syndrome includes non-metabolic features as well [34]. In 2004, the International Diabetes Federation (IDF) made abdominal obesity as a compulsory criterion required for the diagnosis [4]. A joint interim statement of the IDF Task Force on Epidemiology and Prevention, National Heart, Lung, and Blood Institute, American Heart Association, World Heart Federation, International Atherosclerosis Society, and International Association for the Study of Obesity released in 2009 attempted to resolve the disparities between the previous definitions, and to harmonize the diagnostic criteria of MetS. The statement declared that none of the five criteria should be obligatory, and the presence of three out of the following five components qualifies a person for MetS: elevated waist circumference (population- and country-specific definitions), triglycerides (TGs) ≥150 mg/dL (drug treatment for elevated TGs is an alternate indicator), HDL-cholesterol <40 mg/dL in men, <50 mg/dL in women (drug treatment for reduced HDL-cholesterol is an alternate indicator), blood pressure ≥130 mmHg (systolic) and/or ≥85 mmHg (diastolic) (antihypertensive drug treatment in a patient with a history of hypertension is an alternate indicator), blood glucose ≥100 mg/dL (drug treatment of elevated glucose is an alternate indicator) [5].

In fact, due to the reported association between chronic diseases such as T2DM and cardiovascular disease, and the disturbances of the circadian rhythm, recently, Zimmet P et al. (2019) argued that circadian disruption may play a causative role in MetS, and, therefore, proposed the syndrome to be renamed as ‘Circadian Syndrome’ [35].

## 3. Dyslipidemia and Fatty Liver Disease in Animals

Dyslipidemia and fatty liver disease constitute two entities that reflect a profound disturbance in the homeostasis of lipid metabolism. Although they apparently have the same pathological significance, these terms are not synonyms, and define different and successive stages of a deviated lipid metabolism. Dyslipidemia signifies a disturbance in the metabolism of lipoproteins [36,37]. Although dyslipidemia is an infra-clinical disorder, sometimes clinical symptoms belonging to primary diseases responsible for disturbances of lipid metabolism (e.g., hypothyroidism, T2DM, hyperadrenocorticism, systemic inflammatory response syndrome) can be identified. Conversely, the fatty liver disease is a lipid dysmetabolism with a well-defined clinical expression, but with distinct etiopathic peculiarities by species. In most domestic animal species, fatty liver disease occurs either due to decreased nutritional intake (e.g., starvation, anorexia) under normal energy consumption, or due to a sharp contrast between abnormal nutritional intake and increased energy needs imposed by changes in physiological state (e.g., advanced gestation, intense lactation, initiation of laying eggs in laying hens). The severity of the proposed pathogenetic mechanisms may be aggravated by some nutritional imperfections (e.g., choline deficiency, methionine, carnitine, the presence of mycotoxins—aflatoxins), or inadequate maintenance and zoo-hygiene (stress, overcrowding, overheating).

### 3.1. Fat Cow Syndrome in Dairy Cows

In dairy cow medicine, the existence and development of fat cow syndrome (FCS) is not a new issue, and clinical problems associated with this pathophysiology are numerous, but insufficiently understood. FCS was first described in 1976 as a combination of metabolic, digestive, infectious, and reproductive disorders that affect obese periparturient dairy cows, occurring most frequently in loose housing, where these cows are fed and managed together with dry cows disregarding the lactation stages [38]. Emerging data from animals indicate that FCS is a frequent metabolic disorder occurring in commercial farms in heavily lactating dairy cows, especially in the period between three weeks before and three weeks after parturition [39,40]. This ailment can cause considerable economic problems in the dairy industry worldwide. It has been estimated that 40 to 60% of high lactating dairy cows can develop moderate to severe FCS within two to four weeks after calving on an annual basis, and mortality among these cows can reach 25% [39,40,41,42].

Overfeeding of dairy cows during the dry period leads to overconditioning (body condition score >3.5, 5-point scale) [16]. Overconditioning usually starts at late lactation period when milk production is reduced, but dietary energy intake is not reduced properly. High body condition score, negative nutrient balance due to excess feeding during early to late lactation and/or during the dry period, and the accumulation of a large amount of NEFAs are the major risk factors of FCS development [40,43]. Transition from late gestation to early lactation is a dynamic period in dairy cows, and is associated with decreased feed intake, decreased plasma glucose level, and increased plasma lipid and NEFA concentrations [44]. After calving, the feed intake of dairy cows decreases, whereas lactation slowly increases. Due to this physiological process, the body lactose consumption of the cow faces an insufficient sugar supply, blunting the insulin response in specific tissues, and stimulating the mobilization of the fat in the liver [45,46,47]. In comparison with other species, the bovine liver is not able to efficiently export TGs to very-low density lipoprotein (VLDL). Thus, TG are excessively accumulated in the liver, resulting in impaired hepatic metabolisms, and susceptibility to FCS [46,48].

#### 3.1.1. Clinical Signs

**Figure 1:**
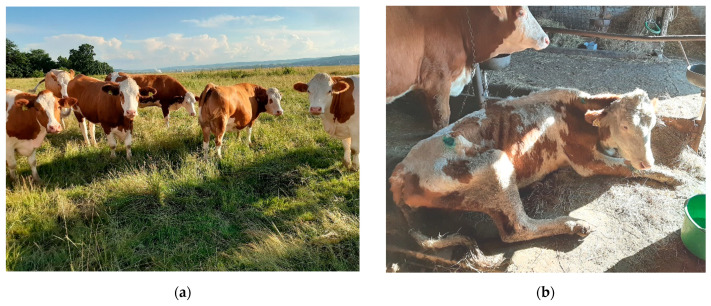
Signs of fatty liver syndrome in dairy cows: (**a**) obese cows during dry period diagnosed with fatty liver syndrome; (**b**) excessive (or rapid) body condition score loss in the second week after calving. Source: authors’ private collection.

FCS is commonly associated with periparturient problems, such as reduced productivity, fertility, and immune functions, or might even lead to liver failure and premature death [43]. Moreover, dairy cows with FCS are prone to suffer of milk fever, ketosis, mastitis, metritis, or retained placenta [49,50,51]. From the clinical point of view, cows affected with milk fever are unable to stand, and progressively lose their consciousness. Ketotic cows often refuse grain before forage, and present reduced milk production, and an apparently empty abdomen, whereas cows with mastitis and metritis experience significant loss of appetite, and produce a low quantity and quality of milk [51]. Dry and almost-dry cows may not present visible signs of obesity at the moment of FCS diagnosis; detection of excess weight might be possible in comparison with herd mates (Figure 1a). During the first few weeks of the post-partum period, the decrease in voluntary feed intake combined with high energy consumption related to milk production generate a negative energy balance. This is clinically expressed by a rapid deterioration of the general body condition, and excessive weight loss (Figure 1b).

#### 3.1.2. Diagnosis

The diagnosis of FCS might be difficult if affected cows are mostly asymptomatic and exhibit no differences in milk production in comparison to their clinically healthy herd mates. Unfortunately, in most of cases, fatty liver is diagnosed post-mortem, or when the cow dies because of other diseases, or it has been culled for being a downer or declared a chronically sick cow. Though some biochemical parameters could provide some diagnostic information [43], a percutaneous liver biopsy is currently the most reliable method to determine the severity of fatty liver in dairy cows. It has high sensitivity, and can be routinely performed at many commercial laboratories, but can be cost prohibitive. Lipolysis can be detected measuring the serum NEFA and β-hydroxybutyrate concentration under laboratory conditions [52], but no test is available for the on-field assessment of NEFA level. Thus, in such conditions, elevated NEFA levels might be identified via correlation with other parameters related to lipid metabolism (increased cholesterol, increased TG, decreased HDL), for which handheld devices are available [53].

#### 3.1.3. Prevention and Treatment

Preventive measures of FCS in dairy cows include the mitigation or the total elimination of the potential risk factors: counteracting oxidative or cytotoxic damage of the liver, bacterial endotoxemia, and ruminal acidosis; and, most importantly, improving the metabolic state of cows in the peripartum period by supplying an extra source of blood glucose, and by decreasing mobilization of NEFA from adipose tissue [43,54]. FCS, if it is not addressed in time, can progress to moderate or even severe forms. Fatty liver is reversible at an early stage, but its treatment highly depends on the extent of lipid infiltration, and the etiology. Proper nutrition can reduce the risk and severity of negative energy balance, which is crucial to prevent the development of the disease in dairy cows. Special attention should be paid to environmental stresses, such as heat and cold stress, high humidity or poor air circulation, inadequate bunk space, housing, free stalls, changing herd mates, and poor sanitary conditions [43,55]. Mild and moderate forms of FCS can be treated by prevention techniques, including the control of feed intake and physical exercises. According to previous studies, providing fresh and high-quality legume or grass hay for feed can increase energy intake [56,57]. Furthermore, moderate daily exercises (1 h/day) can stimulate the oxidation of ketone bodies in the muscle, thus reducing the risk of ketosis [58,59].

Recent evidence suggests that glucose or glucose precursors efficiently reduce plasma NEFAs and the severity of fatty liver at calving, and can be supplied by injections of hormones which may cause an insulin response. The effects of the administration of glucose or glucose precursors orally or as subcutaneous injections have been maximized when large amounts (10% or 50% dextrose IV with slow release at a rate of 60 g per hour, or the equivalent dose two or three times) have been administrated on a daily basis [41,43]. Similar effects can be achieved with glucagon and insulin [57,60]. Propylene glycol delivered as an oral drench (300–600 mL/day) has effectively reduced prepartum plasma NEFA concentration [61]. Glucagon administration (10 mg/day, IV, for 14 days) proved to be effective in reducing liver TG, stimulating glycogenolysis, gluconeogenesis, and insulin production, which ultimately increases the concentration of plasma glucose [43]. Insulin has an antilipolytic effect: it decreases lipid mobilization from adipose tissue, thus preventing the accumulation of TG in the liver. One hundred international unit (IU) intramuscular dose of a 24-h slow-release insulin immediately after calving may be effective, whereas a high dosage of insulin could result in hypoglycemic shock, and should not be used without concurrent glucose administration [60]. The use of glucocorticoids to treat cows with fatty liver is still a subject of various debates due to their potential lipolytic effect, which might increase the NEFA release from adipose tissue, thus promoting the occurrence of ketosis [43]. Other research has revealed that the antioxidant effects of selenium and vitamin E have a crucial role in not only the milk and meat quality produced for human consumption, but also in liver protection [41,62]. As part of fatty liver therapy, cobalt sulfate solution and B complex vitamins were suggested to be administered to improve the low appetite of the affected cows [63]. Basoglu et al. (2002) found that nontoxic dosages of sodium borate improved immune function, or decreased infections, thus, helping to prevent the occurrence of FCS, but further studies should be carried out to strengthen this hypothesis [64]. Choline chloride doses (50–100 g/day) delivered on three consecutive days have also been suggested for the removal of fat from the liver [65]. Other approaches focusing on supplemental fat prepartum, niacin or nicotinic acid administration, or other compounds involved in lipid metabolism proved to be unsuccessful in preventing fatty liver [43,66,67,68].

In summary, the prevention success of FCS in dairy cows highly depends on the nutrition and management program of the farm. The most promising and scientifically proven treatments for mild to severe forms of fatty liver are continuous intravenous infusions of glucose combined with glucocorticoid, insulin injections, or continuous intravenous infusions of glucagon administrated for several consecutive days [43,57,69]. Cows with advanced stage of fatty liver need a more aggressive and long-term treatment.

### 3.2. Comparison between FCS and MetS

In both FCS and MetS, adipose tissue has an important endocrine function. Previous reports have demonstrated that overconditioned cows present IR, and, similar to humans, the adipose tissue can produce different bioactive molecules [70,71,72,73]. It has also been observed that the susceptibility of dairy cows to FCS is also strongly related to a pro-inflammatory state occurring during early lactation, and promotes fatty liver development [74,75]. The immunity of FCS cows is attenuated, and the animals become more susceptible to a variety of diseases [38,76].

Although IR is a more familiar term in association with MetS, it has been suspected to play a key role in the FCS. More accurately, IR appears to be a phenomenon that occurs in most modern, high yielding dairy cows undergoing a negative energy balance due to peak lactation [77]. In comparison with other mammals, the glucose metabolism of ruminants is characterized by low peripheral glucose concentrations, and a low insulin response of the peripheral tissues [47]. Humans with generalized IR and a deficit function of the insulin receptors appeared to have normal blood lipid levels, whereas patients with post-receptor deficiencies expressed signs of dyslipidemia similar to those found in patients suffering from metabolic syndrome [78]. Therefore, it has been suggested that NEFAs are able to interfere with the insulin-signaling pathway triggering IR. Another study suggests that the pathogenesis of IR involves several inhibitory molecules which can interfere with the tyrosine phosphorylation of the insulin receptor and its downstream effectors [79]. Excessive fat mobilization results in an overload of NEFA, and this overload of NEFA surpasses the liver’s capacity to produce apoprotein B and VLDL, especially in ruminants [15]. Though MetS is strongly related to IR and diabetes, the glucose and insulin concentrations in dairy cows at early lactation are low because of the high demand for glucose during milk production [80]. Finding the balance between energy intake (calorie level of the diet) and physical activity can be an effective preventive measure of metabolic dysfunction in humans. In dairy cows, reduced calorie intake is beneficial during the dry period, followed by a progressive increase during the 2–3 weeks antepartum period, and the 3–4 weeks postpartum period that peaks during intense lactation.

Previous studies have demonstrated an expression of mRNA for TNF-α, IL6, MCP1, leptin, adiponectin, visfatin, and resistin in the adipose tissue of dairy cows [58,69,71,72]. In a study conducted in five different peripheral and visceral fat samples from 12 different cows, the m-RNA expression of above-mentioned substances was higher in visceral fat as compared to subcutaneous fat [81]

The association between metabolic syndrome and liver problems has been investigated for a long time in both dairy cows and humans, and it was observed that the liver of affected individuals frequently shows signs of steatosis. Patients with NAFLD presented obesity, T2DM, and dyslipidemia, but there are reports highlighting the development of NAFLD following a rapid weight loss, very similar to those observed in cows [39]. The main cause of developing NAFLD in these patients was the increased level of serum TG, and decreased levels of HDL [82,83]. In dairy cows, there were no significant difference in total cholesterol and HDL-cholesterol concentrations between cows with optimal and adipose body condition [84]. Untreated hepatic steatosis can progress to nonalcoholic steatohepatitis followed by the development of cirrhosis, which, ultimately, can progress to liver cancer. The percentage of patients developing hepatocellular carcinoma after cirrhosis ranges between 4–27% [85]. As in the case of animals, most patients diagnosed with NAFLD do not present specific symptoms that could be attributed to their liver disease. However, available literature data indicate that patients with NAFLD exhibit fatigue or daytime sleepiness, which exerts a negative impact in their life quality and daily activities: 50% of NAFLD-affected patients experienced mild cognitive symptoms, and 46% experienced moderate or severe cognitive impairment [86,87].

## 4. Equine Metabolic Syndrome

The term equine metabolic syndrome (EMS) was first introduced and defined by Johnson in 2002 as an association of obesity, IR, and laminitis [88]. Later, in 2010, the American College of Veterinary Internal Medicine published a consensus statement, and included the following factors into the definition of EMS: increased adiposity in specific locations (regional adiposity) or generally (obesity), IR, and a predisposition towards laminitis. Additional conditions were hypertriglyceridemia or dyslipidemia, hyperleptinemia, arterial hypertension, altered reproductive cycling in mares, and increased systemic markers of inflammation [89]. Recently, in 2019, the European College of Equine Internal Medicine delivered an up-to-date consensus on EMS, and stated that this cluster of risk factors (but not a disease per se) holds ID as a central hallmark where hyperinsulinemia is the most important feature that might occur either as a compensatory response to IR or independently of IR. Also, the presence of obesity is often reported in association with EMS, but it is not mandatory [90]. EMS may be associated with a lean phenotype, and obesity may be present in the absence of IR or EMS. Therefore, the proof of ID existence in an obese animal is compulsory to confirm the diagnosis of EMS. Other features of EMS include increased blood pressure, heart rate, and cardiac dimensions; hypoadiponectinemia; and hyperleptinemia. Though laminitis is the main clinical consequence of EMS, horses with EMS might be at increased risk of hyperlipidemia, hyperglycemia, and hypertriglyceridemia [90].

ID plays a crucial role in EMS and hyperinsulinemia, especially postprandial hyperinsulinemia, which is the most important pathophysiologic feature of ID in horses [91,92,93]. Decreased hepatic clearance of insulin might also provoke hyperinsulinemia, especially in horses with obesity and IR, since more than 70% of the insulin secreted in the pancreatic beta cells is normally cleared from the portal blood by the liver [94]. Field-based observations have highlighted that hyperinsulinemia induced laminitis in horses, which is strongly associated with ID; clinical laminitis has been observed to occur as a consequence of 48–72 h insulin infusion [95,96]. In another study, histopathological evidence of laminitis was experimentally induced by 48 h of glucose infusion, triggering hyperglycemia (mean 11 mmol/L) and endogenous hyperinsulinemia (mean 208 μIU/mL), though none of the treated horses became lame during the experiment [97].

### 4.1. Hyperlipidemias and Hepatic Lipidosis

Equine hyperlipidemias can develop various forms, which is the reason why different terms are also used to differentiate them based on the severity of these diseases. In this context, four terms are currently used, and defined by the level of serum TG concentrations (mg/dL) in the blood of horses: hypertriglyceridemia (serum TG concentration >100 mg/dL, no evidence of clinical disease), hyperlipidemia (concentration between 100 and 500 mg/dL, absence of gross lipemia), severe hypertriglyceridemia (concentration >500 mg/dL, absence of gross lipemia), and hyperlipemia (concentration >500 mg/dL, visible signs of lipemia and fatty infiltration of the liver or multiple organ systems) [98,99,100]. Previous scientific evidences have indicated that concentrations above 1200 mg/dL in the blood can cause death, but their effect highly depends on the type of horses being affected [98,100,101,102,103,104]. It has been proven that hyperlipidemia usually occurs as a result of a negative energy balance predominantly caused by feed restriction, especially during high-energy requirement periods, such as pregnancy, lactation, or disease-induced anorexia [98,103]. However, other findings have suggested that female, obese, and stressed donkeys present the highest risk of developing hyperlipemia regardless of their status of pregnancy [105].

**Figure 2:**
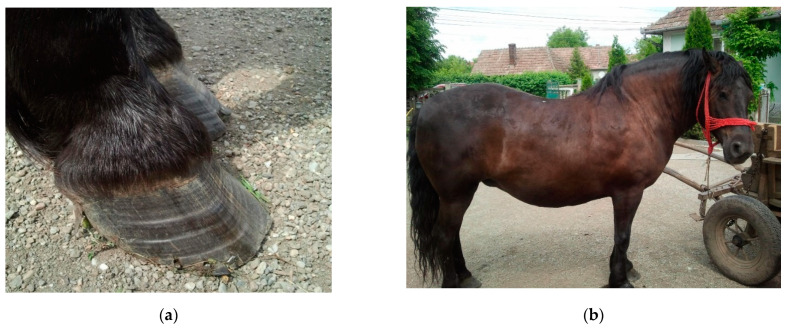
Ex. A 7-year-old Romanian Draft Horse mare with physical characteristics of equine metabolic syndrome and laminitis: (**a**) divergent growth rings in the hoof, indicating laminitis; (**b**) general physical aspect of the horse with EMS. Source: author’s private collection.

There are several factors which can induce abnormalities of energy metabolism in horses, but the most frequent cause is IR. Various investigations have been carried out to demonstrate that equids with IR are predisposed to hyperlipidemia [98]; however, the exact etiology of hyperlipidemia remains unknown. Hyperlipidemia most frequently appears as a primary disease; however, many other experiments and clinical trials were performed to present the existence and development of hyperlipidemia as secondary to various systemic diseases which result in negative energy balance. Among these, disease enterocolitis, dental disease, bacterial infections, pneumonia, colic impactions, parasitism, and laminitis were the most common diseases affecting miniature breeds [100,106,107] (Figure 2a). The list could be continued with hypovolemia, electrolyte imbalances, hepatorenal insufficiency, or esophageal laceration [99,101,108].

#### 4.1.1. Clinical Signs and Diagnosis

**Figure 3:**
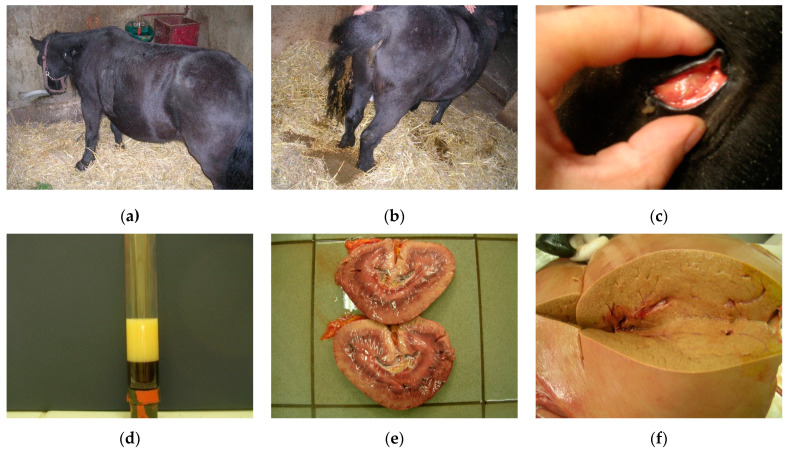
Clinical signs of hyperlipidemias in ponies: (**a**) profoundly depressed pony with hyperlipemia; (**b**) diarrhea as a sign of hyperlipemia; (**c**) hepatic icterus; (**d**) blood sample from a pony with hyperlipemia showing marked opalescence of the plasma; (**e**) lipid accumulation in the kidneys due to hepatic dysfunction; (**f**) damaged fatty liver of hepatic steatosis. Source: author’s private collection.

Clinical signs of hyperlipidemias are non-specific, may vary depending on the degree of metabolic disturbance, and may not be related to the loss of liver function. The most common signs include dullness, lethargy, lack of appetite, decreased water intake, and anorexia. These can progress to severe signs, such as depression, colic, cachexia, and coma (Figure 3a). Affected animals typically experience a rapid loss of body condition, diarrhea, fever, ventral edema (Figure 3b) [98,101,109], and hepatic icterus (Figure 3c). Moreover, animals affected with this disease had glossy blood, and opaque lipemic plasma (Figure 3d). A simple laboratory blood test can reveal whether concentrations of all lipids, especially TG, NEFA, or VLDL, fall into healthy ranges. The results of biochemical analyses performed in miniature horses and ponies diagnosed with hyperlipemia revealed an impaired function of their liver. Though general blood tests can only reveal lipemia, lipemia is not present in all forms of hyperlipidemias. Moreover, lipemia is usually uncommon in large-breed, standard-size horses affected with severe hypertriglyceridemia [99]. For this reason, more reliable confirmation for a definitive diagnosis of hyperlipidemia can be achieved by the analysis of serum TG concentrations, blood glucose, liver enzyme, and even liver biopsy [100].

Hepatic involvement can be detected by the increased concentration of bilirubin, and hepatocellular and biliary enzyme levels [99,104,110,111,112]. Constant and long-term increase in serum TG concentrations triggers lipid accumulation in the liver, kidneys, myocardium, and skeletal muscles, leading to the depletion of these organs, and death (hepatic failure) (Figure 3e). Serum bile acid level measurements can be used to verify hepatic dysfunction, and avoid death.

It is known that horses and ponies possess a remarkable liver capacity to convert free fatty acids into TGs; however, the ketone body formation pathway in ponies are more developed. After hepatic synthesis, TGs are incorporated into VLDL mediated by lipoprotein lipases, and either became available, following hydrolysis, for energy production, or are stored in the adipose tissue. When the liver’s capability to process incoming free fatty acids is exceeded, ponies release large amounts of mobilized lipid back into the plasma [104]. In horses with hyperlipidemia, hyperactivity of lipoprotein lipases was found to result in an excess production of VLDL. Under stress conditions, massive mobilization of fatty acids from fat tissue is induced as an exaggerated response to the action of catecholamines [113]. In ponies with hyperlipidemia, the activity of lipoprotein lipase, which is responsible for peripheral catabolism of TG, is doubled than that in healthy ponies, likely as a physiological response to the increased concentration of substrate. The largest fraction of TG produced by the liver in ponies with hyperlipidemia is a buoyant fraction of VLDL [104]. In pony breeds, the overproduction of TGs may be precipitated in IR during stress periods.

The cumulative effect of excess VLDL and lipomobilization results in dyslipidemia and fatty liver disease (Figure 3f). Unlike ponies, horses benefit from a highly efficient liver system of TG synthesis and incorporation into VLDL. In addition, lipomobilization under the effect of catecholamines is significantly lower in horses than in ponies, and does not reach the critical points for the development of dyslipidemia and liver lipidosis. Although there are reports of sporadic cases of natural hyperlipidemia in horses, the observed clinical symptoms (inappetence, lethargy, depression) have been attached to primary inflammatory organopathies [99].

#### 4.1.2. Prevention and Treatment

Various approaches have been proposed to prevent the development of hyperlipidemias in equids, but the most effective measures are the reduction of stress-inducing factors (stress during transport, changes in management conditions, or parasites) and negative energy balance [98,99]. Stress factors can be controlled by appropriate attention and management [106,107]. In miniature horses, donkeys, ponies, and horses with systemic disease associated with hypophagia and high metabolic demands, nutritional supplementation can prevent hyperlipemia [109]. Another possible way to prevent the development of hyperlipidemia in susceptible animals might be to improve their insulin sensitivity. Since there is a vast amount of evidence on the linkage between obesity and ID, dietary restriction could be an effective solution in order to reduce body weight in predisposed animals [114]. However, special attention should be attributed to this practice because inadequate reduction of energy intake could provoke a negative energy balance and, consequently, hyperlipidemia [115]. Fat feeding could improve the TG clearance in ponies, but the diet might lead to impaired glucose tolerance and IR [116,117]. Conversely, other studies have found that IR and adiponectin concentrations can be decreased more efficiently by a regular nonstructural carbohydrates-rich diet, rather than forage or fat-rich diets [118,119,120]. However, the association of dietary nonstructural carbohydrates intake and insulin regulation is not so obvious; some recent work has indicated that though the adaptation to high nonstructural carbohydrate diets can improve glucose tolerance and tissue insulin sensitivity, it could lead to an exaggerated postprandial insulinemic response [91,121].

Effective management for treating hyperlipidemias in horses can be reached only if the disease is identified in early stages, and the therapy is started before triglyceridemia becomes severe. The first and most important step is to recognize the animals with a predisposition for hyperlipidemias. After recognition, the correction of the underlying diseases and conditions that caused the negative energy balance are of a great importance. Nutritional supplementation is one of the essential factors that needs to be corrected in the treatments of hyperlipemia. In fact, nutritional support can reverse the existing negative energy balance, increase serum glucose concentrations, stimulate endogenous insulin release, and inhibit the mobilization of peripheral adipose tissue [98,109]. In cases where the affected individual refuses voluntary feed intake, enteral nutrition becomes necessary. Enteral nutritional support is the most natural nutrient delivery approach, making the intestinal mucosa to be partially dependent on the digestion products for energy and nutrients. Positive responses to oral administration of small amounts of simple sugars or high-fructose corn syrup have been reported with a dosage of 5 kcal/kg/d administrated multiple times per day [98]. Other results have shown that enteral nutritional supplementation and treatment of the primary disease often reversed hyperlipemia in miniature horses and donkeys, but less frequently in ponies [109]. If the enteral route of administration is not available for various reasons (gastric reflux, bowel distention, ileus), parenteral administration remains the only solution. However, this is expensive and poses the risk of secondary complications, including hyperglycemia, hypertriglyceridemia, thrombophlebitis, and an increased risk of bloodstream infections [122]. Parenteral solutions with added lipids might be used in horses that need long-term support, or those unable to tolerate carbohydrates [123]. In more severe cases, when more complex nutritional supplementation is required, nasogastric intubation should be used.

Pharmacological attempts have also been carried out to find potential agents to avoid the development of hyperlipidemias, particularly in IR horses. Results have shown that metformin and a synthetic thyroid hormone called levothyroxine can improve insulin sensitivity by controlling blood glucose concentrations through the inhibition of hepatic gluconeogenesis and glycogenolysis [124,125,126,127]. These agents have successfully been used in human medicine as an insulin-sensitizing agent, but their effectiveness in horses is controversial. When administrated orally at high dosage, levothyroxine induced weight loss in horses and reduced intestinal glucose absorption and insulinemic responses to oral carbohydrate ingestion [93,124,126]. Pretreatment of clinically healthy horses with levothyroxine for 14 days prevented the development of IR following endotoxin infusion [93,98].

Hyperlipidemias can also be treated by insulin therapy, but patients with IR are more difficult to treat, and need special care to prevent hypoglycemia. Subcutaneous administration of insulin can result in periods of hypoglycemia post-administration, but this effect can be alleviated by oral carbohydrates given in parallel. To determine the appropriate dose of insulin and carbohydrates for treatment, blood glucose levels need to be determined first. Insulin therapy for the treatment of hyperlipidemias is still a subject of scientific debates. On one hand, Moore et al. (1994) declared that insulin administration did not improve the medical stage of the affected animals, and proved to be inefficient because the excessive fat mobilization occurred as secondary to IR [104]. On the other hand, Durham et al. (2008), and Waitt et al. (2009) reported that insulin administrated simultaneously with adequate nutritional support alleviated hypertriglyceridemia in equids [124,128]. As an alternative, heparin has been tested and used in the treatment of hyperlipidemias in horses due to its stimulative action upon peripheral utilization of triglycerides and lipoprotein lipase activity. However, the efficiency of this treatment was questioned by other researchers who obtained negative results: heparin administration may induce bleeding complications, thrombocytopenia, and pancreatitis [100,103,129].

### 4.2. Comparison between EMS and MetS

MetS differs from equine disease, as the most important pathologies that occur are T2DM and cardiovascular diseases in humans, and laminitis in horses. In both humans and equids, high energy diet combined with sedentary lifestyle have resulted in obesity and metabolic disturbances. Moreover, the adipose tissue does not only serve as fat storage, but also has endocrine functions in both species. The adipose tissue produces adipokines (adiponectin, leptin, visfatin), and releases inflammatory mediators and pro-inflammatory cytokines [130]. Though mechanisms behind these processes are less documented in equids as compared to humans, the adipose tissue inflammation that affects metabolic and biochemical processes show similarities between both species [131,132,133,134]; metabolic syndrome has been associated with chronic systemic inflammatory state, and concentrations of inflammatory cytokines have been correlated with obesity and IR [130,132,135]. Pro-inflammatory cytokine expression, including IL-6 and TNF-α, have been detected in the lamellar tissue of horses with hyperinsulinemia [136]. Elevated levels of circulating NEFAs have also been associated with IR and physiological disturbances in several tissues [113,137,138,139,140].

Though hypertriglyceridemia and low HDL-cholesterol concentrations are criteria used to define MetS, obesity and onset of IR are not associated with decreased HDL neither in equids nor cows. VLDL and HDL-cholesterol concentrations are usually greater in obese and IR horses as compared with healthy horses, and this is likely to be attributable to an increased activity of lipoprotein lipases [141]. In the study by Frank et al. (2006), plasma NEFA, VLDL, and HDL-cholesterol levels were 86%, 104%, and 29% greater, respectively, in obese IR horses than in non-obese horses [142]. In humans, the transfer of cholesterol from HDL to VLDL is catalyzed by cholesterol ester transfer protein, a protein that is not active in equine blood [143,144].

In pathological conditions associated with overproduction of cortisol, catecholamines, and somatotropic hormone, lipolysis is stimulated by the elevated activity of hormon-sensitive lipases, but it is accompanied by reduced activity of lipoprotein lipases in both equids and cows. The conjugate effect of these enzymes results in lipomobilization coupled with a decreased liver capacity to convert NEFAs to TGs, and incorporate TGs in VLDL, leading to a significant increase in serum NEFA concentration. Thus, elevated serum NEFA levels indicate both a marked degradation of lipids in adipose tissues and liver dysfunction. Consequently, the assessment of NEFA is more important for the diagnosis and evaluation of lipomobilization syndrome in horses and cows than the determination of lipoproteins.

General obesity can accompany ID in equids with EMS. Horses and ponies with EMS should be examined for various evidence of laminitis, such as divergent hoof growth rings, or third phalanx rotation. Increased adiposity is evident before laminitis in most animals; however, it has been observed that not all obese horses present ID and laminitis, and lean phenotypes are not necessarily an exception to this rule [145]. Similarly, in human medicine, there is a group of obese people who have metabolically healthy obesity, featured by non-dysfunctional adipose tissue with higher adiponectin levels, lower inflammatory markers, and different fatty acid composition [146]. These people do not present ID, and have a supposedly more favorable prognosis for cardiovascular disease, cancer, and all-cause mortality as compared with obese and insulin-dysregulated individuals [146,147,148]. However, if obesity is not addressed in time, a considerable proportion of these individuals could develop ID and MetS [149].

MetS affects all organ systems involved in the metabolism, and is characterized by abdominal obesity, hypertension, dyslipidemia, IR, vascular dysfunction, and inflammation of adipose tissue [150]. Clinical signs of EMS include general or regional adiposity, IR, and increased oxidative burst [151,152]. MetS and obesity have also been associated with neuropathies, such as distal symmetric sensory polyneuropathy, which is a major risk factor for the development of diabetic foot syndrome, accompanied in many cases by peripheral arterial occlusive disease (atherosclerosis) [153,154]. Previous studies conducted on laminitic horses suggested that neuropathic changes are rare, but have an important contribution to pain response in this disease. However, until now, no direct relationship between laminitis-associated neuropathy and EMS or ID has been described, even though diabetes in humans has been used as a comparison together with other causes of neuropathy [155,156,157].

In humans, IR induces increased secretion of insulin by the pancreatic beta-cells to regulate glucose concentration in blood. However, compromised beta-cell function results in hyperglycemia and glucotoxicity [158]. Conversely, equids with EMS have compensated hyperinsulinemia. The development of hyperinsulinemia in horses is highly dependent on dietary composition, which is an important factor in the development and management of MetS as well. It has been observed that horses fed on a high cereal diet became obese, and presented ID, whereas horses fed on a high fat diet became obese, but without alterations of insulin sensitivity [118]. Similarly, a high fat diet was associated with higher insulin sensitivity as compared to a high starch and sugar diet [120]. In a human diet, partial replacement of digestible carbohydrates by unsaturated fats in the daily diet may positively affect the metabolic processes in obese individuals [159]. Simple sugars in a human diet are known to contribute to IR and obesity, whereas unsaturated fats have been proven to improve metabolic profiles, and decrease inflammation [160,161]. However, further investigations are needed to better understand the effect of dietary composition on insulin metabolism and inflammatory mediators in horses, which could help to improve dietary management [162]. Negative energy balance is more difficult to control in equids than in humans, especially in female individuals, since their pregnancy and lactation periods automatically alter their energy balance. Previous clinical results have shown that early enteral feeding has a positive effect not only in humans, but also in horses [98,163,164,165,166]. Physical exercises are efficient and safe practices to reduce body weight and adiposity, and have shown some insulin sensitivity stimulating effects as well [167]. Therefore, the optimal prevention technique to avoid hyperlipidemias is likely to set up a very well balanced plan combining dietary restriction with physical activity, similar to that proposed for human patients.

T2DM is a major problem in people with metabolic syndrome, and is less likely in equids, though horses may also develop diabetes in some cases of EMS [168]. In humans, the most common risk factors associated with T2DM are a family history of diabetes, hypertension, impaired glucose tolerance, being overweight, physical inactivity, ethnicity, and poor nutrition during pregnancy [169,170]. Risk factors in horses include metabolic syndrome and PPID, but available scientific evidence is scarce [171,172].

## 5. Conclusions

Metabolic syndrome in humans differs in several aspects from both the cow and equine disease. The most important pathological factor is the affliction of the cardiovascular system in humans; fatty liver in cows; and the development of laminitis in horses. The mechanisms that lead to these potentially life-limiting consequences are not fully comparable, although the changes in these species take place in the vascular system. Inflammatory conditions in adipose tissue, and the effects on metabolic and biochemical processes show similarities between all species. There are still many underlying mechanisms of metabolic syndrome which were not scientifically exploited. Therefore, the current comparative approach might serve as a starting point for future investigations and improvements in this field.
